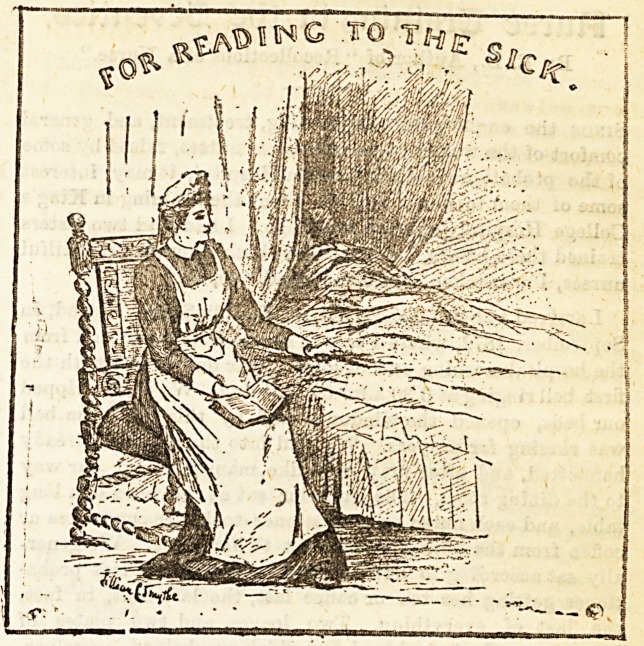# The Hospital Nursing Supplement

**Published:** 1891-05-16

**Authors:** 


"The Hospital" Nursing Mirror.

Being the Extra Nursing Supplement of "The Hospital" Newspaper.

Contributions for this Supplement should be addressed to the Editor, The Hospital, 140, Strand, London, W.C., and should have the word "Nursing" plainly written in left-hand top corner of the envelope.

### En Passant.

NURSES FOR NORTHALLERTON.—On May 6th, the Hon. Mrs. Dundas presided at a meeting at Northallerton for the establishment of a district nurse. She said that the meeting was called to promote the Rural Nursing Association—a branch of which had been established in Northallerton—whose object it was to have trained nurses available in cases of need in the rural districts. They hoped by training nurses in their hospitals in time to attain a degree of efficiency which would enable them to become affiliated to the Queen's Nursing Institution. Mrs. A. F. Godman read the rules of the Northallerton Rural Nursing Association, and also a list of the ladies and gentlemen in the neighbourhood who had subscribed. She also stated that Mrs. Wharton, of Skelton Castle, had established an association, which she was wishful to affiliate with their Association.

ANOTHER WARNING.—A letter in a Liverpool paper with regard to the death of Nurse M'Arthur, says: "It would not be a bad idea to warn the nurses against that recklessness which too often springs from association with danger. I attended the patient from whom I suspect Miss M'Arthur caught the fever. He was a bad case certainly. Being unable to comprehend me when I spoke to him, I got Nurse to put a question to him, and was certainly taken aback at the fearless way in which she placed her face close to his mouth whilst he was gasping out deadly pestilence. I spoke to one or two of them afterwards, and warned them of the danger; but they only laughed, and said they were not afraid. To be sure, we know that fearlessness is one of the best safeguards against contagion; but at the same time it is possible to put one's head into the lion's mouth once too often."

SHORT ITEMS.—Nurse Mary Mills, the first district nurse at Hartlepool, is proving popular and efficient.—Dr. Allan Gray, in his report of the irregularities at Leith Hospital, mentions the over-work of the nurses; there are other serious points in the nursing question there that need remedying.—The district nursing started at Worthing by Miss Twining has been so successful that another nurse is to be engaged.—Dr. Ewart, of Colney Hatch, has, we are glad to hear, arranged a course of St. John's Ambulance lectures for the attendants.—Miss Manning, Matron of Coventry City Hospital, has collected subscriptions for a piano for the nurses' room, and a handsome instrument has been obtained.—At the suggestion of Lady Londonderry, a committee has been formed at Stockton to provide trained nurses for the poor.—Miss Tillet is lecturing on nursing at the Portsmouth Hospital.

THE COUNTESS OF ZETLAND.—Many, very many, are the friends of influence who now take an intelligent interest in nurses and their needs, and amongst them one of the most gracious and charming is Her Excellency the Countess of Zetland. Ever since Lady Zetland went into residence at the Viceregal Lodge, Dublin, she has devoted much of her time to visiting hospitals and nursing institutions. She has been good to the patients and kind to the nurses. On the 30th ult. Lady Zetland was "at home" from four to seven to the nurses of the Adelaide, Meath, Orthopædic, and other hospitals. The day was fine, and the Countess and her daughters received their guests in the garden; pleasure-boats were supplied on the lake, and a sumptuous tea provided. Needless to say, the nurses thoroughly enjoyed this pleasant change from their trying labours, and went away rested in mind and body.

THE SECOND THOUSAND.—The nurses are joining the Royal National Pension Fund so fast at present that upwards of one hundred new policies were applied for last month. Already 946 proposals towards the second thousand have been received, but as some sixty of these may not be accepted, some 110 nurses have still an opportunity of being included if they make application at once. Assuming the second thousand is completed during the present month, arrangements will probably be made for their reception by our gracious President in July next.

NURSES' HOME.—The Princess Christian has most kindly established and opened a Home of Rest for Nurses at Brighton. In an account of the proceedings sent us by a correspondent it is stated "the Institution is in no way connected with the Royal British Nurses' Association"; but, as another report states, "Upon her arrival Her Royal Highness was introduced by Dr. Bedford Fenwick, and was presented with a beautiful bouquet by Mrs. Bedford Fenwick on behalf of the British Association of Trained Nurses, which institution claims the honour of having employed the first trained nurse that ever worked in Brighton," we have our doubts whether reports are to be trusted. Certainly there were trained nurses in Brighton years before the British Nurses were either heard or dreamed of. The *Brighton Guardian* says: "On account of the lack of funds it has only been found possible to furnish a sufficient portion of the house to allow for the accommodation of half the number of nurses that might otherwise be received.

WANTS AND WORKERS.—We have received a large number of queries and demands lately, some of them very extraordinary. Several times we have been able to supply addresses of old friends to our readers, several times we have been able to put two readers in communication, one of whom wanted to be rid of what the other desired. So comes the thought, Why not a new column of wants and workers? Thus, under "Wants"—The nurses' bed-rooms at the Co-operation, 8, New Cavendish Street, would be improved by some prettily illuminated lines, which would be gladly received by the Lady Superintendent. Then under "Workers" we might get this: Miss J., of Wandsworth, is willing to illuminate lines for any institution desiring them. Many of our readers are not nurses, but ladies interested in nursing and willing to help hospitals in any way they can; we are sure many of them would send magazines or papers, or make flannel jackets for any institutions in need of the same. Many a nurse's home could be enlivened by pictures and books, many a poor patient's wardrobe re-stocked by those to whom the work would be a pleasure. It is terrible to think of the many idle young ladies in London longing to lend a helping hand, wanting above all things to be useful and beloved, and on the other side so much work with no one to do it. Not long since a nurse wanted to get an orphan boy into an asylum; she had to send out hundreds of applications for votes, and this she did in her two short hours off duty. Surely there must have been plenty of idlers who would willingly have undertaken this work had they known. Then think of the secretaries sending out appeals and annual reports; think of some of the wards that have no visitors to read to the patients, no friend in the country to send them flowers! Shall we start our new column, readers? Shall we try and be the means of pointing out to those noble hearts to whom the career of a nurse is denied a hundred other ways in which they can help? Family ties, ill-health, youth, and other reasons restrain the would-be nurse, but none the less there is outlet for her generous enthusiasm if she knew it. We will watch this week and see if we get lists of "wants" and lists of "workers.'

### Lectures on Surgical Ward Work and Nursing.

By ALEXANDER MILES, M.B. (Edin.), C.M., F.R.C.S.E.

### LECTURE XXIV.—SAYRE'S JACKET AND STARCH SPLINTS.

USES.—This application is used most largely in curvature of the spine due to disease of the vertebræ. It may be augmented by applying extension, either to the lower limbs with the foot of the bed raised, or to the upper part of the spine from the axilla, in which case the head of the bed will be raised. When the disease is in the cervical region extension may be applied to the head; and when in the middle of the back it may be applied both to the head and legs.

CAUTION.—It is of the very first importance that the nurse should understand the rationale of the treatment of disease of the spinal column, especially when that is situated in the cervical region. The great danger is that the ligaments which protect the spinal cord from pressure by the bones

*By kind permission of Messrs. Weiss and Sons.*

should become softened and useless, and that during some movement of the patient's head or body the diseased bones will become displaced and press on the cord. In the lower part of the canal this would cause paralysis, but in the upper cervical region instant death would be the result. This has not infrequently been the result of the nurse moving the patient against the doctor's orders.

MATERIALS REQUIRED.—1 to 7, same as for plaster case; 8, a quantity of absorbent wool; 9, semmet made of boracic lint; 10, tripod for suspending patient. The bandages should be broader then for a case for limb. The semmet should not fit very closely, and should extend a few inches below the plaster. It is in such cases that the toxic action of boracic acid on fleas, &c., is most valuable.

METHOD OF APPLYING JACKET.—Having thoroughly washed the skin with soap and water, all bony prominences such as the vertebral spines and iliac crests should be carefully protected with pads of antiseptic wool or of boracic lint. "If the patient be a female, and especially if she be developing at the time, it will be necessary to apply a pad under the shirt over each breast before the plaster bandage is put on. These pads should be removed just before the plaster sets, and at the same time pressure should be made over the sternum for the purpose of indenting the central portion of the plaster jacket, and of thus giving form to the body and removing pressure from the breasts." (Sayre.) Sayre uses, in addition, what he calls a "dinner pad." This is a pad put over the region of the stomach, and removed just as the plaster is setting. The space thus left permits of the dilation of the stomach after food. The suspension apparatus is used so that the spinal column may be rendered as straight as possible when the jacket is being applied, in the hope that this position will be maintained by the rigid apparatus. A child may be held up by the arms, but in an adult this is obviously impossible. The apparatus consists of a tripod, from the centre of which hangs "a curved iron cross beam, to which is attached an adjustable head and chin collar with straps, and also two axillary bands." The patient is raised by the head and axilla till the toes just touch the ground, and in this position the jacket is applied. The bandages, which are prepared and applied in exactly the same way as those used for a plaster case, only differ in being somewhat broader. After applying a plaster-jacket or case, you may find some difficulty in removing the plaster from your hands and nails. This will be obviated if you add a handful of sugar to the water, as plaster of paris is soluble in a saccharine solution.

TO REMOVE A PLASTER CASE OR JACKET.—This is a thing which requires to be done with the greatest possible care, as any sudden or excessive movement, such as might result from roughly trying to remove the plaster, may undo all the good the application has accomplished, as, for example, in excision of the knee or after fractured leg the union may be broken down. Various methods have been suggested for accomplishing your object without force, such as dissolving the plaster with strong hydrochloric acid poured into a narrow scratch made in the plaster, cutting it through with bone pliers and a strong knife, or with a short, strong saw. Whichever method you adopt, be particularly careful never to use any force whatever without having thoroughly fixed the part which was to have been kept at rest.

### STARCH BANDAGE.

USES.—This appliance is used to fix on light splints, such as pasteboard or poroplastic, where the main object is to fix the joint rather than to give it support. Starch is rarely used alone as a splint, because it is not sufficiently strong and takes a long time to set—about twelve hours. Its great advantage over plaster of paris is that it is much lighter.

MATERIALS REQUIRED.—(1) A quantity of ordinary household starch (Glenfield); (2) bowl and spoon; (3) kettle of boiling water; (4) jug of cold water; (5) supply of ordinary cotton bandages; (6) boracic bandages; (7) pasteboard or poroplastic splints; (8) scissors.

METHOD OF PREPARING THE MATERIAL.—Starch.—First break down about six ounces of starch with cold water till it is of the consistence of a thick paste, and white in colour. Then add boiling water, and stir thoroughly till the starch assumes a blue tint, and becomes so fluid that it pours freely from the spoon. As it cools it sets into a jelly, and it may either be used in the state of jelly or in its fluid state before cooling. The addition of one part of powdered boracic acid to nine parts of starch is an advantage. Splint.—Cut the poroplastic to the required length and breadth, and then having moistened it in hot water, carefully mould it to the exact shape of the part after all the necessary padding has been applied.

MODE OF APPLYING THE MATERIALS.—The limb having been thoroughly washed with soap and water, cover it in from the extremity towards the trunk with a boracic lint bandage of appropriate size. Carefully pad all bony prominences, and other parts where it is undesirable to have pressure. The splint having been accurately moulded to the shape of the limb thus protected, is fixed by means of an ordinary cotton bandage. Over this smear a coating of the starch, and rub it thoroughly into the bandage. Another layer of bandage is applied over this, and covered over with starch in the same way; and so on till three or four layers have been applied. The starch takes about twelve hours to dry thoroughly, and until it has done so no weight may be borne by it, as it will yield and be useless. Placing the part between hot bottles or in front of a fire will hasten the drying process.

### Work in the Holy Land.

I HOPE that some readers of THE HOSPITAL will be interested in the small beginnings of a medical mission to the Druzes of Baakleen, Mount Lebanon.

Directly we went to live among them, intending to help in the schools, they came to us with all their sufferings from illness or accident. With the help first of a native doctor, and latterly of a trained nurse and dispenser, spared to us from Mr. Meredith's Mission at Jerusalem, we have done what we can, but we feel continually what a great boon a Cottage Hospital would be. Miss Wordsworth Smith is only able to spare one room of her house for the Dispensary, so that many people have to wait in a kind of verandah or in the washhouse, and if any case needs a serious operation and nursing afterwards, we cannot help except by sending them down to the Prussian Hospital at Beyrout, ten hours' ride on a mule! Baakleen is a good centre for the mountains north and east of Sidon.

The people often show much gratitude for the help and sympathy afforded to them when suffering. Those who can are asked to pay a trifle for their medicines, which are only given freely to the very poor, for they are apt to throw away bitter medicine if they have paid nothing for it. Those who can pay nothing sometimes bring offerings of milk, eggs, or fruit. One very small girl brought a jar of water from the distant fountain, it being the only thankoffering within her power.

Practical people will remark, "It will not do to build a hospital where water is so scarce." I am pleased to tell you that, after six years of talk on the subject, the Druzes of Baakleen have united to bring water in iron pipes from a spring higher up the mountains, and it is very good wholesome water. Besides this a hospital ought to have a reservoir for rain water.

One day a baby girl was brought to us with half her face in a fearful state from a burn, smeared over with some native medicine, and one eye closed up; we feared it was destroyed. The mother had gone out leaving her on the floor, and her two-year-old brother had kicked her against the fire, which was also on the floor. Can you not imagine our pleasure when, after several days' dressing, that eye opened, bright as the other one!

Our object is not only to treat their suffering bodies, but also to make known the Saviour, so the Dispensary work generally begins with a Scriptural address from our native doctor, or from our Biblewoman. The writer of an otherwise admirable paper upon the Druzes in *Blackwood's Magazine* says: "One could fearlessly challenge any missionary to produce a genuine case of a convert from Druzedom who has arrived at years of maturity." Who will help us to accept this challenge?

One rough man, when told that his wife was likely to die, said, "Never mind, I can soon get another wife; will a few days settle it?" By this mail we hear that he is better in action than in words. He has actually washed and dressed this awful leg twice a day, and the last time that he was visited he said that if his wife recovered he should believe in Christ. If any are willing to help, will they communicate with Miss Lloyd, 143, Clapham Road, S.W.?

### Death in Our Ranks.

WE announce with regret the death of one of the nursing staff of the Withington Hospital. Nurse Maria Helen Chorlton, 24 years of age, contracted scarlet fever with serious throat symptoms one week ago. Her illness terminated fatally on Friday night. Nurse Chorlton had just finished the second year of her training. She is deeply regretted by nurses and patients.

### SOWING AND REAPING.

FOR every one of us there is seed time and harvest, and true to nature, as we sow, so shall we reap. We learn from the highest authority and also our own experience, that men cannot gather grapes from thorns, or figs from thistles, so if we are always planting seeds of death in our constitutions by intemperance or excesses of any kind, we must not be surprised if we find ourselves weak and miserable creatures.

Health is one of the greatest blessings God gives to man, and we should do our part also and be very careful that our bodies are kept clean and pure and strong.

The great German, General Von Moltke, who has lately passed away from the midst of us, said he owed his long life of over ninety years to the grace of God and temperate habits. That is the secret worth knowing. We are free agents, and must help to our utmost His work in the world.

The great Sower never fails to cast good seed into our hearts. He has been letting it fall on the world from the beginning of time, and will continue His bounty to the end. "All through the centuries the victory of every nation, of every individual soul has been only this, the victory of the unwearied generous sowing of the heavenly seed," says a modern writer. "God from eternity could not be satisfied until He had given every creature the good seed which might bring forth fruit thirty, sixty, or a hundredfold."

Shall we then by our careless actions, our sloth or love of pleasure, choke the good seed and prevent its growing up into "fair deeds of charity" which are the fruits of the spirit of love?

When our hearts get hardened by worldliness or self-love, or self-indulgence, God will sometimes mercifully break up the fallow ground for us by trials of various kinds, so that they become softened and receive with gratitude the pity and kindness lavished on us by our Saviour and His followers, drinking them in like soft showers on a thirsty land which strengthen and refresh at the same time. Then, having received largely without upbraiding from our Maker, we will freely and thankfully give to our neighbours the sweet blossoms of gentleness and meekness, in the love which thinketh no evil, but hopeth all things, endureth all things.

We thank thee, oh, our Father,
For all things bright and good,
The seed time and the harvest,
Our life, our health, our food;
Accept the gifts we offer
For all thy love imparts,
And, what thou most desirest,
Our humble, thankful hearts.

### Nurse Training in the Seventies.

By E. D., Author of "Recollections of a Nurse."

Since the enquiry into the feeding, treatment, and general comfort of the women to be trained as nurses, raised by some of the probationers of the London Hospital, it may interest some of them to know a little about the training in King's College Hospital eighteen years ago. I had had two sisters trained there before, and as they both turned out skilful nurses, I was taken under the usual age, 25.

I arrived at Saint John's House, Norfolk Street, Strand, in September, 1872, where I was to sleep, walking to and from the hospital morning and evening. Our day began with the first bell ringing at 6.30 a.m. for dressing. We then stripped our beds, opened the windows, and by this time the bell was ringing for prayers. We filed into the oratory, already bonnetted, and after prayers in like manner found our way to the dining-room. The Superior sat at the head of a long table, and each nurse or probationer took her cup of tea or coffee from the Superior and went to her place. We generally sat according to our length of service, the last probationer getting her tea or coffee last, the last seat, in fact, the last of everything. Two loaves and two plates of butter graced the table, from which we helped ourselves. The nervous way in which the new probationers handled that bread and butter used to make us older ones quake, only a very short time being allowed for this meal, and if you did not look sharp you went to the hospital pretty hungry. Somehow this meal, more than any other, always reminded me of the flight of the children of Israel out of Egypt. Haste, haste, haste seemed to be written upon everything. Directly after breakfast you made your bed and tidied your corner of the room, and then started to walk to the hospital, about a quarter of a mile, to be on duty by a quarter to eight. My introduction into hospital life was as a probationer in the largest female medical ward of K.C.H., containing twenty-four beds. There was a Sister, nurse, assistant nurse, and myself as probationer. My work was to wash everything that was washable on the ward table; change the water in flower vases, see that clean towels were ready for the doctors, and clean the pewter inkpot; when this was done I put on a large saucepan of milk to boil, another of beef tea, then went round to the patients with basins and bread. By this time the milk and beef-tea were ready, and I gave each patient some, then I cleaned the two saucepans, collected the basins, washed them and put them away; then I filled the boiler ready for fomentations and poultices.

During this time the Sister was generally looking over her report received from the night nurse; the nurse and assistant-nurse washing and in other ways attending to the worst cases. At 9.30 the house-physician, accompanied by some of the students, would go round to every patient, the sister and nurse being there to remove poultices, give the night report, answer any questions, and receive the doctor's instructions for the day. As the fomentations and poultices are removed for sounding and other purposes, the assistant-nurse is ready with another to put on at once, the probationer helping, so that by the time the house-physician has finished with his last patient, the ward is already clear and orderly. Dinners for the patients come up at twelve o'clock in large square hot-water trays, each patient's dinner separate, except the potatoes, which come in a net unpeeled, and the probationer sets to work upon these at once. The rice or sago pudding comes up in a tin dish and is served out to each patient. When they have all finished the plates are collected and taken to the day room to be washed by the scrubber, the probationer taking a bowl of water to wash each patient's knife and fork; then she has to sweep the ward. During this time the Sister has gone to her dinner, and the assistant nurse; the nurse giving special feeds to the worst cases, or watching a delirious case. When the other two have returned the nurse and probationer go. This meal generally lasted twenty minutes, and consisted of roast or boiled joints and vegetables. No sooner than you had finished your dinner the visiting physicians came, and there was a repetition of the morning work. Every other day the probationer goes off duty from 2 until 4 o'clock. We nearly always spent the time lying down to rest our weary limbs. Of course this did not answer; we began to look pale, so a rule was made that unless you went out for a walk a fine of sixpence would be imposed, and as none of us had many sixpences to spare we went out. I generally walked alone to the little churchyard of the Savoy at the back of the Strand, there I sat until it was time to go back to the hospital to prepare the patients' tea at 4.30. Most of them had an egg either beaten up with milk or boiled, and bread and butter with cocoa or tea for those who were allowed it. Then you wash up the tea things, and from five until six o'clock the tea is going on for the nursing staff. As this was the only meal where talking was allowed, or where we had time to talk, you may imagine its being a favourite one with women. It consisted of bread and butter, now and then a cake; besides this we sometimes got friends to send us a few country eggs, which we cooked upstairs and carried down in our pockets, only the first two or three times I so carried them I had the misfortune to sit down before taking them out. After this chatty meal you went to the wards and received instructions in the art of cleaning heads, washing feet, and giving patients a bath. At 7.30 you heat the milk and beef tea, and take it round as you do in the morning; remove and wash all basins. At eight, or soon after, prayers are read in the ward, then you clear up the day room and start for St. John's House at a quarter to nine p.m. Our supper at the Home was rather like the breakfast, tea or cocoa, a dish of slices of cold meat, of which you were helped to one, or you could have a slice of bread pudding, but not both. You had your supper, like your breakfast, bonneted, and then filed out to prayers; from the oratory to your bedroom. Now most folks would think the work was over. Not so; the gas was turned to its highest pitch and garments waved this way and that way to spy out the little creatures who come to us during the lesson upon the art of cleaning heads. Blistered feet are another great hindrance to getting to sleep quickly, which nearly all probationers suffer from at first, to say nothing of the feeling of emptyness after these meagre suppers. But out goes the gas, and it seems only a few minutes before the bell rings to get up.

*(To be continued.)*

### Appointments.

Nurse Sarah Carter of the Withington Hospital has just been appointed nurse at the Kendal Union Infirmary.

Nurse Elizabeth Munro and Nurse Miriam Toft were appointed charge nurses at Withington Hospital on Wednesday. They have each taken temporary charge of wards for some time.

The Santa Claus Home.—Miss R. Napper, for many years Matron of the Princess Alice Hospital at Eastbourne, has been appointed Matron of the new Convalescent Home for Children to be opened shortly at Highgate. The Home is in connection with the Santa Claus Society, and is chiefly for cases of hip and spinal disease; girls will be received in the Home up to fourteen years of age, boys up to ten years of age. We wish the Home every success.

### Everybody's Opinion.

*[Correspondence on all subjects is invited, but we cannot in any way be responsible for the opinions expressed by our correspondents. No communications can be entertained if the name and address of the correspondent is not given, or unless one side of the paper only be written on.]*

### A PRO.'S GRIEVANCES.

"E. de G." writes: Having had good experience in training schools, I cannot allow "Poor Pro.'s" letter to pass without some comment, as I consider what she says casts a great slur on "one of our largest training schools in the North of England," and I cannot help thinking she has been misinformed. It is true that in most infirmaries "pros." are first placed for one or two months in "crib" or infirm wards, in order to teach them the first essentials of nursing, viz., keeping patients clean. This also helps to weed out those who are unfit for nurses, as if they find this trying part of nursing too much for them they are unworthy to go on with the work. I myself was trained and had charge of various wards in a large infirmary, and I have often found that girls who are unfit for nursing grumble because they are first of all given minor duties to perform. They cannot expect to "run before they can even crawl." One probationer I knew once told me that before she came to the hospital she had pictured herself "holding a leg for the doctors to amputate" the day after her arrival, and felt much injured when she was sent to the infirm and simply to wash patients, rub backs, and comb heads, &c. Surely there is no hospital where mothers with infants only three days old are indiscriminately mixed up with other patients. Then, again, it seems to me to indeed be strange management for nurses working in the maternity wards to be allowed to do general nursing, as the fear of infection is at these times so great. Besides, when in the lying-in wards I always found there was ample work for all four of the nurses in training there, and when we had time to sit down we were expected to study. Certainly we were never allowed even to visit the other wards in the hospital. If such a state of things exists as "Poor Pro." points out, the quicker it is seen into the better, as many lives must now be risked. "Poor Pro." forgets all about night duty, and this always forms a parts of a nurse's training.

### PRIVATE NURSING.

"E. M." writes: Will you kindly allow me, through your paper, to say that I quite agree with all Nurse Laura quotes as her experience of eighteen months? I have myself been engaged for six years and a-half as a private nurse, during which time I have been into seventy different homes, and have attended patients of nearly every rank, and can honestly assert that, with very few exceptions, I have always met with the greatest kindness and consideration. There are, I am willing to admit, some unreasonable persons in the world, but I do not think it any harder for a nurse to come in contact with them sometimes than a poor domestic servant or tradesman, and certainly no nurse is compelled to remain if she cannot get ordinary necessaries. If we who intend to take up private nursing as a profession would try not to think too highly of our professional value, but simply feel when we go to a patient that our duty is to comfort and relieve them as much as possible, and use the knowledge we have acquired by our training for that purpose, not forgetting, at the same time, that a little kind consideration and sympathy is often needed and generally appreciated by the relatives and friends, we should, I think, also be quite ready to make allowances for any discomfort that might exist in the household arrangements, and then I am sure that, as a rule, we should be treated as true friends instead of being looked upon as persons to be dreaded, as I know we are by a great many. On the other hand, if a nurse on her arrival tries to impress every one with her professional knowledge, requires a great deal of attention and gives a lot of unnecessary trouble, no wonder if she finds herself disliked. My opinion is, that a private nurse's life may in most cases be a very useful and happy one.

### An Old Prescription.

Professor Boerhave and Osterdike's Regimen prescribed for the Gout. A.D. 1750.—We are of opinion that the gout is not to be cured by any other means but by a milk diet, which will in twelve months' time alter the whole mass of the blood; and in order thereto, the following directions must be strictly observed and followed:—

I. You must not taste any liquor, only a mixture of one-third milk and two-thirds water, your milk as new as you can get it. A little tea and coffee is also permitted, with milk.

II. In the morning as soon as awake, and the stomach has made a digestion, you must drink eight ounces of spring water, and fast two hours after; eat milk and bread, milk pottage, or tea with milk, with a little bread and fresh butter.

III. At dinner you must not eat anything but what is made of barley, oats, rice, or millet seed, carrots, potatos, turnips, spinage, beans, peas, &c. You may likewise eat fruit when full ripe, baked pears or apples, apple dumplins; but above all, milk and bisket is very good, but nothing salt or sour, not even a Seville orange.

IV. At supper you must eat nothing but milk and bread.

V. It is necessary to go to bed betimes, even before nine o'clock.

VI. Every morning before you rise to have your feet, legs, arms, and hands well rubbed with pieces of woollen cloth for half an hour, and the same going to bed. This article must be strictly observed, for by this means the humours, knobs, and bunches will be dissipated, and prevent their fixing in the joints, by which they become useless.

VII. You must accustom yourself to exercise, the more the better; but take care of the cold weather, winds, and rain.

### Notes and Queries.

To Correspondents.—1. Questions or answers may be written on post-cards. 2. Advertisements in disguise are inadmissible. 3. In answering a query please quote the number. 4. A private answer can only be sent in urgent cases, and then a stamped addressed envelope must be enclosed. 5. Every communication must be accompanied by the writer's full name and address, not necessarily for publication. 6. Correspondents are requested to help their fellow nurses by answering such queries as they can.

### Queries.

(9) *A Gift Offered.*—Would any nursing institution like to have the monthly parts of The Hospital a fortnight after publication?—*Miss R.*

(10) *A Gift Wanted.*—Where can I obtain a letter for the Brompton Chest Hospital for a very deserving case?—*R. Hart*, 99, *New Road, E.*

### Answers.

*Donati.*—There is plenty of work for a masseur in Melbourne if he goes out with good introductions and good certificates and does not give himself airs. Colonial people are rather touchy, and unwilling to acknowledge that any good thing can come out of England.

*R. Hart.*—We have put your request as a query above, and possibly one of our readers may send you a ticket. Failing this, get a list of subscribers to the hospital from the Secretary, and write to, or call on, those nearest to you.

*A. M. G.*—Thank you for particulars. Please send the other cases you mention; the lack of experience is quite as dreadful as the lack of training.

### Presentation.

The Committee of the Barton-on-Humber District Nursing Association recently presented to Miss Alice Dannath, the hon orary Secretary and Superintendant, a handsome walnut-wood Davenport, fitted up with all writing requisites. The following letter accompanied the gift:—"Dear Miss Dannath,—Feeling that you have given a great deal of time and thought as Secreta ry to our Nursing Association, and that it is greatly owing to your help and experience that the district nursing has been so successful, we hope you will accept this little Davenport in acknowledgment of all your kind work."

### The Invalid Children's Aid Association.

### II.—WHAT THE ASSOCIATION WANTS.

THE chief needs of the Association are visitors to go and see the little invalids, money to provide surgical appliances, instruments, invalid carriages; money's worth in the shape of clothing, old or new, linen rags (all the better for being softened with much washing and wearing); games, toys, books, materials for fancy work, such as knitting, or any occupation which can be carried on by invalid children, either for their own benefit or that of others.

All these things will be useful for the children in their own homes, but there are cases where the nature of the complaint, or the conditions of a child's home, make his recovery a hopeless matter, unless he can be put into fresh circumstances. It is the saving of many a little paralysed or ricketty child if he can be sent for some months to a place where he will get good air, nourishing food, and proper care. Letters for convalescent homes are most useful in helping such cases, or money to pay for the boarding out of invalids in healthy country places, and good Nursing Homes. This is an expensive part of the work, as the treatment sometimes takes a long time, or no permanent result can be looked for.

There was one case of a little ricketty boy, who was twice sent away, but each time, from some unforeseen circumstance, he had to come home again, just as he was beginning to put his feet to the ground. The consequence was, that each time he went back again, till at last the Association arranged for his being away some months at a stretch, and now he is able to run about as a child ought to do. There are also the cases of spinal curvature, where a child may be ordered to lie down for a year or two. In some poor homes, where there is a very large family, or where the mother is the breadwinner and there is no one to wait upon the child, it is practically impossible to carry out the treatment; it is not done, and the child's back grows worse and worse. In a good Nursing Home the child is made to keep in one position, is carefully watched and tended, and if he can remain there long enough there is every chance of the curvature being cured. This involves considerable expense, but money can hardly be better bestowed than in helping a child to grow up healthy and vigorous instead of sickly and deformed.

Where the children can be helped in their own homes, visitors are much wanted to undertake individual cases, especially visitors who will go to children in out-of-the-way places. There are so many things a kindly and sensible visitor can do or suggest for an invalid child's comfort: sometimes she can put an inexperienced mother in the way of attending better to the child herself; sometimes she can show her how to obtain the services of a trained nurse—district, or otherwise—where the child has wounds which need dressing, or surgical appliances which need adjusting, with more skill than the mother has at command. Another visitor will bring a fresh interest into a dull and melancholy little life by teaching some deformed or crippled child to read. It is such a universal practice for the children of the poor to be taught at school, that a child who must be kept at home is apt to be quite uninstructed. Other children, who are too young to care for reading, or whose state of health might forbid them the exertion, may be given toys or games, and shown how to use them; beads to thread, wool and canvas for mat-making, the many devices for occupying little fingers sold at kindergarten shops, pictures to cut out, scrap-books to look at, and scrap-books to make, transparent slates with a nice lead pencil fastened to one corner of them by a bit of string, picture-books and story-books will each and all help to wile away many a weary hour. Any fresh suggestions and appliances for occupying invalid fingers would be gladly welcomed at the office in Buckingham Street. Where invalid children are concerned one wants a large choice of occupations, so as to ensure something suitable for every class of infirmity; also one must not expect from an invalid the same perseverance and steadiness of effort one looks for in a healthy child, and occupations must be varied to prevent fatigue of body or lassitude of mind.

Old toys and old books often answer the purpose as well as, or better than, new ones; and it is best that the books should not be too large or heavy for children to hold comfortably. Where a visitor has a family of children at home she will find the best incentive to carefulness in her own nursery will come from proposing to her children to keep their toys and books in good order with a view to passing them on to the invalid children when they have done using them themselves.

Old clothing and new are alike valuable; flannel jackets, vests, and night dresses being especially useful. But, above all, personal service is needed, kind hearts and active brains, which will busy themselves with the task of making the lives of the poor little invalids more comfortable and bearable than they are at present. The address of the Association is 18, Buckingham Street, Strand, London, W.C.

### What to Read.

A VERY helpful little book lately published is "Blessed be Drudgery," which can be had in brown boards for 1s. 2d. It can be had for a shilling in white paper covers at all the book-stalls, but these pretty white covers soil so quickly. "The Little House on the Cliff" is a charming shilling volume published by the R.T.S.; it is a story about children, by M. B. Manwell, who always writes well about the little ones. There is some clever word painting in "A New England Nun, and Other Stories," by Mary E. Wilkins, to be had at all the circulating libraries. Cardinal Newman's "Apologia" and Froude's "Life of Carlyle" are proving the most popular volumes of the cheap Silver Series published by Longmans. Grant Allen's "The Tents of Shem" has just come out as a two shilling yellow-back; it is a good companion for a railway journey.

### Amusements and Relaxation.

### SPECIAL NOTICE TO CORRESPONDENTS.

### Second Quarterly Word Competition commenced April 4th, ends June 27th, 1891.

Competitors can enter for *all* quarterly competitions, but no competitor can take more than *one first prize* or *two* prizes of any kind during the year.

Proper names, abbreviations, foreign words, words of less than four letters, and repetitions are barred; plurals, and past and present participles of verbs, are allowed. Nuttall's *Standard* dictionary only to be used.

N.B.—Word dissections must be sent in WEEKLY not later than the first post on Thursday to the *Prize Editor*, 140, Strand, W.C., arranged alphabetically, with correct total affixed.

The words for dissection for this, the SEVENTH week of the quarter, being

"EXCURSION."

| Names. | May 7th. | Totals. | Names. | May 7th. | Totals. |
|---|---|---|---|---|---|
| Christie | 5 | ... 176 | Ivanhoe | | ... |
| Patience | 5 | ... 172 | Weta | 5 | ... 147 |
| Agamemnon | 6 | ... 178 | Lady Betty | 5 | ... 169 |
| Hope | 6 | ... 179 | Mortal | — | ... 76 |
| Reldas | 6 | ... 175 | Little Eliza | 5 | ... 147 |
| Lightowlers | 5 | ... 167 | Dove | — | ... 95 |
| Nurse J. S. | 6 | ... 135 | Ladybird | 6 | ... 141 |
| Qu'appelle | 5 | ... 170 | Psyche | 5 | ... 148 |
| Jenny Wren | 5 | ... 163 | Ugug | 5 | ... 120 |
| Wyameris | 6 | ... 174 | Harrie | — | ... 18 |
| Paignton | 5 | ... 147 | Grannie | 5 | ... 129 |
| Theta | 5 | ... 170 | Eale | 4 | ... 129 |
| Success | — | ... 17 | Grimalkin | — | ... 53 |
| Tired | | ... | Nurse G. P. | 4 | ... 4 |
| M. G. | 5 | ... 153 | | | |

**Notice to Correspondents.**

N.B.—All letters referring to this page which do not arrive at **140, Strand, London, W.C.**, by the *first post on Thursdays*, and are not addressed PRIZE EDITOR, will in future be disqualified and disregarded.

## Figures and Tables

**Figure f1:**
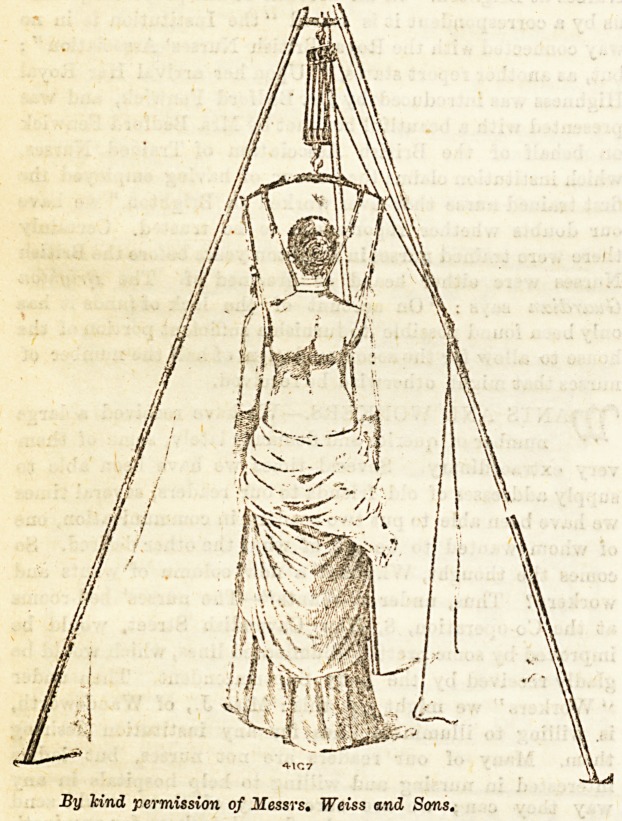


**Figure f2:**